# Socioeconomic characteristics and comorbidities of diverticular disease in Sweden 1997–2012

**DOI:** 10.1007/s00384-017-2853-1

**Published:** 2017-08-07

**Authors:** Maziar Nikberg, Jianguang Ji, Jerzy Leppert, Kristina Sundquist, Abbas Chabok

**Affiliations:** 1 Department of Surgery, Västmanland’s Hospital Västerås, SE-721 89 Västerås, Sweden; 2 Centre for Clinical Research of Uppsala University, Västmanland’s Hospital Västerås, SE-721 89 Västerås, Sweden; 3 Center for Primary Health Care Research, Department of Clinical Sciences, Lund University, Lund, Sweden; 4 Department of Medicine, Västmanland’s Hospital Västerås, SE-721 89 Västerås, Sweden

**Keywords:** Diverticular disease, Socioeconomic status, Education, Income, Comorbidity

## Abstract

**Purpose:**

This study aimed to evaluate the association of socioeconomic status and comorbidities with uncomplicated and complicated diverticular disease (DD) in Sweden.

**Methods:**

We identified all individuals aged ≥30 years in Sweden diagnosed with DD between 1997 and 2012 using the Swedish National Population and Housing Census and the Hospital Discharge Register. Data were analyzed by multivariable logistic regression, with individual-level characteristics as covariates.

**Results:**

A total of 79,481 patients (median age 66 [range 30–86] years) were hospitalized for DD, 15,878 (20%) of whom for complicated DD. Admissions for both uncomplicated and complicated DD were more common in women (*p* < 0.001). A low education level was identified as a risk factor for uncomplicated (unadjusted hazard ratio [HR] 1.79, 95% confidence interval [CI] 1.75–1.82; adjusted HR 1.22, 95% CI 1.19–1.24) and complicated DD (unadjusted HR 1.84, 95% CI 1.77–1.92; adjusted HR 1.26, 95% CI 1.21–1.32). Patients with the lowest income had a lower risk of hospitalization for uncomplicated (adjusted HR 0.94, 95% CI 0.91–0.96) and complicated DD (adjusted HR 0.87, 95% CI 0.83–0.92) than those with the highest income. The correlation coefficient between income and education was 0.25. Diabetes and cardiovascular disease were identified as protective factors against uncomplicated DD (adjusted HR 0.68, 95% CI 0.66–0.69 and HR 0.79, 95% CI 0.74–0.84, respectively).

**Conclusions:**

Patients with the lowest education level had an increased risk of hospitalization for DD. Further studies are needed to explore the association of diabetes and cardiovascular disease with uncomplicated DD.

**Electronic supplementary material:**

The online version of this article (doi:10.1007/s00384-017-2853-1) contains supplementary material, which is available to authorized users.

## Introduction

The clinical burden of colonic diverticular disease (DD) is impressive. It is responsible for 312,000 hospital admissions per year in the USA alone, with the incidence of acute diverticulitis increasing and the average age of the patients declining. The severity of the disease varies, and DD presents with a wide spectrum of symptoms ranging from acute uncomplicated to complicated DD [1].

The etiopathogenesis of DD is not well clarified. Epidemiological studies have analyzed the associations between DD and obesity, smoking, alcohol intake, and physical activity [2, 3]. In addition, comorbidities and the use of specific medications can influence the risk of DD. Nonsteroidal anti-inflammatory drugs and corticosteroids have been shown to be a risk factor for complicated and uncomplicated DD, while cardiovascular disease has been found to be a risk factor for complicated DD [3–5]. Socioeconomic variables such as education, income, and access to healthcare have been shown to influence the severity of a range of medical conditions [6–10]. Socioeconomic characteristics such as education, income, and urbanization, together with comorbidities and in addition to known risk factors such as sex and age, may also explain the risk of hospitalization for both uncomplicated and complicated DD.

The relationship of socioeconomic characteristics and comorbidity with disease is of special interest in Sweden, where access to healthcare is equal for all residents, and differences in socioeconomic status (SES) are less apparent compared with many other countries. The aim of this study was to evaluate the association of SES and comorbidities with uncomplicated and complicated DD in Sweden.

## Methods

This study was approved by the Ethics Committee of Lund University, Sweden. All individuals ≥30 years of age living in Sweden in 1997 were included. The data sources used included the Swedish National Population and Housing Census, the Total Population Register, and the Hospital Discharge Register. Access to these registers was provided by Statistics Sweden and the National Board of Health and Welfare. The Swedish Hospital Discharge Register, which was created by the National Board of Health and Welfare in 1964, contains hospital discharge records for all residents of Sweden. From 1987 onwards, the register includes complete nationwide data with information on dates of admission and discharge, together with principal causes of hospitalization [11]. Data in these registers were linked using individual lifetime personal identification numbers assigned to all people living in Sweden. These numbers were replaced with serial numbers to maintain anonymity.

## Outcome variable

DD was identified from the Hospital Discharge Register according to the tenth revision of the World Health Organization International Classification of Diseases (ICD-10 from 1997 onwards). Only principal diagnoses of DD were considered to ensure high validity. Complicated DD was defined as ICD-10 codes K572, K574, and K578, where uncomplicated DD was defined as ICD-10 codes K573, K575, and K579.

## Predictor variables

The SES variables, individual disposable income and years of education in 1997, were identified from the Total Population Register. Individual disposable income was categorized as lowest, middle-low, middle-high, and highest according to the quartile range. Years of education were categorized as 0–9, 10–11, and ≥12.

## Adjustment variables

Adjustments were made for sex, age (30–39, 40–49, 50–59, 60–69, 70–79, and 80+), geographic region of residence (large cities, mid-sized cities, and small cities), and comorbidities (inpatient diagnosis of cardiovascular disease, diabetes, rheumatoid arthritis [RA]/systemic lupus erythematosus [SLE], and chronic obstructive pulmonary disease [COPD]). Comorbidities were defined by the following ICD-10 codes: I00–I99 (cardiovascular diseases), M32, M05, and M06 (RA/SLE), E10–E14 (diabetes), and J40–J47 (COPD). Geographic region of residence was included to adjust for possible regional differences in hospital admissions. Large cities were defined as cities with a population of >200,000 and comprised the three largest cities in Sweden: Stockholm, Gothenburg, and Malmö. Small cities were defined as cities with a population of <90,000.

## Statistical analysis

Cox regression models were applied to calculate hazard ratios (HRs) and 95% confidence intervals (CIs) for admission to hospital with a diagnosis of uncomplicated or complicated DD. A multivariable Cox regression analysis was performed including the selected demographic characteristics (age, sex, and geographic region of residence) and comorbidities (cardiovascular disease, diabetes, RA/SLE, and COPD). All of these confounding factors were defined at baseline. We censored individuals (that is, we treated them as no longer under observation or at risk of the study outcome) at the time of death from any cause, at the end of the follow-up period (December 31, 2012), or at the time of emigration. Data are accurate to two decimal places. A multivariable Cox regression analysis stratified by age was also performed with a cutoff of 65 years. The temporal trend for DD was calculated separately for different age groups. We also tested the polychoric correlation between income and education. All analyses were performed using SAS version 9.2 (SAS Institute, Cary, NC, USA).

## Results

Of 79,481 patients admitted to hospital with a diagnosis of DD, 15,878 (20%) were admitted for complicated DD. The median age for patients with both uncomplicated and complicated DD was 66.0 (range 30–86) years. The age distribution for uncomplicated and complicated DD was the same for both sexes. Admission for uncomplicated (64%) and complicated (61%) DD was more common in women than in men (*p* < 0.001) (Table 1).

**Table 1:** Uncomplicated and complicated diverticular disease in Sweden between 1997 and 2012

Subtype	Uncomplicated		Complicated		Total
	No.	%	No.	%	No.
Total	63,603	1.19	15,878	0.30	5,325,811
Age (years)					
30–39	6567	0.54	1560	0.13	1,220,826
40–49	11,529	0.97	2797	0.24	1,189,676
50–59	14,952	1.31	3908	0.34	1,137,815
60–69	13,444	1.71	3522	0.45	786,630
70–79	13,167	1.88	3155	0.45	701,505
80+	3944	1.36	936	0.32	289,359
Sex					
Men	22,654	0.87	6175	0.24	2,602,304
Women	40,949	1.50	9703	0.36	2,723,507
Education (years)					
≤9	27,538	1.43	6969	0.36	1,927,780
10–11	19,664	1.21	4895	0.30	1,622,085
12+	16,401	0.92	4014	0.23	1,775,946
Income					
Lowest	12,943	1.02	3070	0.24	1,272,026
Middle-low	16,836	1.28	4157	0.32	1,318,012
Middle-high	17,518	1.28	4468	0.33	1,365,639
Highest	16,306	1.19	4183	0.31	1,370,134
Region					
Large cities	8808	1.03	2391	0.28	851,816
Mid-sized cities	21,876	1.20	5356	0.29	1,826,708
Small cities	32,919	1.24	8131	0.31	2,647,287
Cardiovascular disease					
No	49,116	1.21	10,784	0.27	4,058,948
Yes	14,487	1.14	5094	0.40	1,266,863
Diabetes					
No	62,674	1.20	15,553	0.30	5,226,323
Yes	929	0.93	325	0.33	99,488
Rheumatoid arthritis/systemic lupus erythematosus					
No	62,960	1.19	15,484	0.29	5,296,627
Yes	643	2.20	394	1.35	29,184
Chronic obstructive pulmonary disease/asthma					
No	62,095	1.19	15,252	0.29	5,227,515
Yes	1508	1.53	626	0.64	98,296

Cardiovascular disease, RA/SLE, and asthma/COPD were more common in patients with complicated than in those with uncomplicated DD (32 vs. 23%, 2.5 vs. 1%, and 3.9 vs. 2.9%; *p* < 0.001 for all) (Table 1).

In the univariable analysis, a low level of education was identified as a risk factor for uncomplicated and complicated DD (HR 1.79, 95% CI 1.75–1.82 and HR 1.84, 95% CI 1.77–1.92, respectively). Those with the lowest income had a lower risk of hospital admission for uncomplicated and complicated DD (HR 0.89, 95% CI 0.87–0.92 and HR 0.82, 95% CI 0.79–0.86, respectively) (Table 2).

**Table 2:** Hazard ratios of uncomplicated and complicated diverticular disease by socioeconomic status

Subtype	Uncomplicated						Complicated					
	Unadjusted			Adjusted			Unadjusted			Adjusted		
	HR	95% CI		HR	95% CI		HR	95% CI		HR	95% CI	
Education (years)												
12+	1.00	Reference		1.00	Reference		1.00	Reference		1.00	Reference	
10–11	1.34	1.31	1.37	1.24	1.21	1.27	1.36	1.31	1.42	1.27	1.22	1.33
≤9	1.79	1.75	1.82	1.22	1.19	1.24	1.84	1.77	1.92	1.26	1.21	1.32
Income												
Lowest	0.89	0.87	0.92	0.94	0.91	0.96	0.82	0.79	0.86	0.87	0.83	0.92
Middle-low	1.16	1.14	1.19	1.03	1.00	1.05	1.12	1.07	1.17	0.98	0.94	1.03
Middle-high	1.12	1.09	1.14	1.02	1.00	1.04	1.11	1.06	1.16	1.00	1.96	1.04
Highest	1.00	Reference		1.00	Reference		1.00	Reference		1.00	Reference	
Age (years)												
30–39				1.00	Reference					1.00	Reference	
40–49				1.84	1.79	1.90				1.81	1.70	1.93
50–59				2.60	2.52	2.68				2.55	2.40	2.71
60–69				3.74	3.63	3.86				3.34	3.14	3.56
70–79				5.34	5.17	5.51				4.04	3.78	4.32
80+				5.72	5.49	5.97				4.33	3.98	4.73
Sex												
Men				1.00	Reference					1.00	Reference	
Women				1.51	1.49	1.54				1.36	1.32	1.40
Region												
Large cities				1.00	Reference					1.00	Reference	
Mid-sized cities				1.13	1.10	1.16				1.01	0.96	1.06
Small cities				1.14	1.12	1.17				1.02	0.98	1.07
Cardiovascular disease												
No				1.00	Reference					1.00	Reference	
Yes				0.68	0.66	0.69				1.14	1.10	1.18
Diabetes												
No				1.00	Reference					1.00	Reference	
Yes				0.79	0.74	0.84				0.96	0.86	1.08
Rheumatoid arthritis/systemic lupus erythematosus												
No				1.00	Reference					1.00	Reference	
Yes				1.53	1.42	1.66				3.60	3.26	3.98
Chronic obstructive pulmonary disease/asthma												
No				1.00	Reference					1.00	Reference	
Yes				1.13	1.08	1.19				1.74	1.60	1.89

In the multivariable logistic regression analysis, age was identified as the strongest independent risk factor for both uncomplicated and complicated DD. The correlation between income and education in Sweden was tested using the polychoric correlation test, which gave a correlation coefficient of 0.25. Forty-one percent of individuals in the middle-high and highest income categories in Sweden had the lowest level of education. For both uncomplicated and complicated DD, female sex (HR 1.51, 95% CI 1.49–1.54 and HR 1.36, 95% CI 1.32–1.40), RA/SLE (HR 1.53, 95% CI 1.42–1.66 and HR 3.60, 95% CI 3.26–3.98), COPD/asthma (HR 1.13, 95% CI 1.08–1.19 and HR 1.74, 95% CI 1.60–1.89), and low level of education (HR 1.22, 95% CI 1.19–1.24 and HR 1.26, 95% CI 1.21–1.32) were associated with increased risks. Residency in small cities was found to be a risk factor for uncomplicated DD (HR 1.14, 95% CI 1.12–1.17). Patients with the lowest level of income had a lower risk of hospitalization for uncomplicated or complicated DD than those with the highest level of income (HR 0.94, 95% CI 0.91–0.96 and HR 0.87, 95% CI 0.83–0.92). Cardiovascular disease was identified as a protective factor against uncomplicated DD (HR 0.68, 95% CI 0.66–0.69), but as a risk factor for complicated DD (HR 1.14, 95% CI 1.10–1.18). Diabetes was identified as a protective factor against uncomplicated DD (HR 0.79, 95% CI 0.74–0.84) (Table 2).

In an additional multivariable logistic analysis stratified according to the general Swedish retirement age of 65 years, the identified risk factors including income did not differ from the results of the main analysis (Table S1).

## Discussion

This population-based study based on the Swedish national inpatient register showed that patients with the lowest level of education had an increased risk of being hospitalized for DD. The most important risk factor for both uncomplicated and complicated DD was identified as increased age. Interestingly, patients with the highest income level had an increased risk for DD compared with those with the lowest income level, which was unexpected because a higher education level is usually associated with higher income. Because the retirement age in Sweden is 65 years, a separate multivariable analysis was performed to investigate the discrepancy between income and the risk of DD. However, in this analysis, higher income was still associated with an increased risk. This discrepancy could be explained by the low correlation (0.25) between high education and high income found in the general Swedish population.

A study from the USA reported that patients with a low SES were more often admitted to the emergency department with a diagnosis of DD, but it was unclear whether this DD had a complicated history [6]. Furthermore, the subcategories of SES were not presented. Substantial structural differences exist in health care between the USA and Sweden. In a previous study of a Swedish cohort focusing on migration, where SES was defined according to a classification used by Statistics Sweden that is based on occupation but also considers the educational level of the head of household’s occupation, type of production, and position at work, SES did not influence the risk of hospitalization for DD [12]. That study used ICD 9 to identify patients diagnosed with DD between 1991 and 1996 [12]. In ICD 9, in contrast to ICD10, it is not possible to distinguish uncomplicated from complicated DD. Furthermore, no adjustments were made for income or comorbidities. In the present study, when the analyses were adjusted for comorbidities, low education was still found to increase the risk of admission for both uncomplicated and complicated DD.

Patients with asthma/COPD and RA/SLE were found to have an increased risk of admission for DD, especially complicated DD. As previously suggested, this increased risk is probably because of treatment with corticosteroids and immunosuppression [13, 14]. Patients with diabetes have been reported to present with more advanced DD [15]. However, in the present study, diabetes was not predictive of more severe DD, and surprisingly, both diabetes and cardiovascular disease were associated with a reduced risk of uncomplicated DD. Although one can speculate that medication or lifestyle changes may explain the protective effect of cardiovascular disease in uncomplicated DD, the mechanism of this effect is unknown and cannot be explained by the increased use of statins by patients with cardiovascular disease [16].

The retrospective design of the present study has some inherent limitations. Patients were included at their first admission with DD, and subsequent admissions were not considered. Furthermore, because of the large sample size (5.3 million), even small differences between the study groups may be statistically significant. However, the study does have several strengths, including the large number of patients who were diagnosed by clinicians and the use of ICD-10 coding, which is more precise compared with ICD-9. The diagnosis of DD is not always based on explicit criteria, but the increased use of CT in the acute setting should improve diagnostic accuracy. Another important key strength in the assessment of SES status is the avoidance of self-reporting. The novelty of this study is that we had access to SES measures individually linked with almost complete clinical data over a long time period.

## Conclusion

The results of the present exploratory population-based study using the national inpatient register suggest that patients with the lowest level of education have an increased risk of being hospitalized for both uncomplicated and complicated DD. Future studies are needed to verify and explore the association between diabetes and cardiovascular disease with uncomplicated DD.

## Electronic supplementary material

Table S1 (DOCX 20 kb)
