# Supplementary material for: Socioeconomic characteristics and comorbidities of diverticular disease in Sweden 1997–2012
Source: Int J Colorectal Dis. 2017 Aug 7;32(11):1591–6. doi: 10.1007/s00384-017-2853-1 (PMC5635093; doi:10.1007/s00384-017-2853-1)
Supplement: Supplementary file 1 — (DOCX 20 kb) [file 384_2017_2853_MOESM1_ESM.docx]

| **Supplementary Table S1**. Hazard ratios of uncomplicated and complicated diverticular disease by socioeconomic status stratified according to retirement age at 65 years | | | | | | | | | | | | | | | |
| --- | --- | --- | --- | --- | --- | --- | --- | --- | --- | --- | --- | --- | --- | --- | --- |
| Subtype | Uncomplicated | | | | | | |  | Complicated | | | | | | |
|  | < 65 | | |  | ≥ 65 | | |  | <65 | | |  | ≥ 65 | | |
|  | **HR** | **95% CI** | |  | **HR** | **95% CI** | |  | **HR** | **95% CI** | |  | **HR** | **95% CI** | |
| Education (years) |  |  |  |  |  |  |  |  |  |  |  |  |  |  |  |
| 12+ | 1.00 | Reference | |  | 1.00 | Reference | |  | 1.00 | Reference | |  | 1.00 | Reference | |
| 10–11 | 1.27 | 1.24 | 1.30 |  | 1.13 | 1.08 | 1.18 |  | 1.36 | 1.31 | 1.42 |  | 1.11 | 1.01 | 1.21 |
| ≤ 9 | 1.22 | 1.19 | 1.26 |  | 1.14 | 1.10 | 1.19 |  | 1.32 | 1.25 | 1.38 |  | 1.08 | 0.99 | 1.17 |
|  |  |  |  |  |  |  |  |  |  |  |  |  |  |  |  |
| Income |  |  |  |  |  |  |  |  |  |  |  |  |  |  |  |
| Lowest | 0.92 | 0.89 | 0.95 |  | 0.95 | 0.91 | 1.00 |  | 0.91 | 0.85 | 0.96 |  | 0.83 | 0.76 | 0.91 |
| Middle-low | 1.00 | 0.97 | 1.03 |  | 1.05 | 1.00 | 1.09 |  | 0.99 | 0.93 | 1.05 |  | 0.98 | 0.90 | 1.06 |
| Middle-high | 1.01 | 0.99 | 1.04 |  | 1.03 | 0.98 | 1.07 |  | 1.00 | 0.95 | 1.05 |  | 1.00 | 0.93 | 1.09 |
| Highest | 1.00 | Reference | |  | 1.00 | Reference | |  | 1.00 | Reference | |  | 1.00 | Reference | |
|  |  |  |  |  |  |  |  |  |  |  |  |  |  |  |  |
| Age (years) |  |  |  |  |  |  |  |  |  |  |  |  |  |  |  |
| 30–39 | 1.00 | Reference | |  |  |  | |  | 1.00 | Reference | |  |  |  | |
| 40–49 | 1.83 | 1.77 | 1.88 |  |  |  |  |  | 1.80 | 1.69 | 1.91 |  |  |  |  |
| 50–59 | 2.54 | 2.46 | 2.61 |  |  |  |  |  | 2.49 | 2.34 | 2.65 |  |  |  |  |
| 60–64 | 3.30 | 3.18 | 3.42 |  |  |  | |  | 2.93 | 2.72 | 3.16 |  |  |  | |
| 65–69 |  |  |  |  | 1.00 | Reference | |  |  |  |  |  | 1.00 | Reference | |
| 70–79 |  |  |  |  | 1.30 | 1.26 | 1.34 |  |  |  |  |  | 1.11 | 1.05 | 1.18 |
| 80+ |  |  |  |  | 1.35 | 1.29 | 1.40 |  |  |  |  |  | 1.15 | 1.06 | 1.25 |
|  |  |  |  |  |  |  |  |  |  |  |  |  |  |  |  |
| Sex |  |  |  |  |  |  |  |  |  |  |  |  |  |  |  |
| Men | 1.00 | Reference | |  | 1.00 | Reference | |  | 1.00 | Reference | |  | 1.00 | Reference | |
| Women | 1.54 | 1.51 | 1.57 |  | 1.48 | 1.44 | 1.52 |  | 1.26 | 1.21 | 1.31 |  | 1.62 | 1.53 | 1.72 |
|  |  |  |  |  |  |  |  |  |  |  |  |  |  |  |  |
| Region |  |  |  |  |  |  |  |  |  |  |  |  |  |  |  |
| Large cities | 1.00 | Reference | |  | 1.00 | Reference | |  | 1.00 | Reference | |  | 1.00 | Reference | |
| Mid-sized cities | 1.15 | 1.12 | 1.19 |  | 1.10 | 1.06 | 1.14 |  | 1.04 | 0.98 | 1.11 |  | 0.99 | 0.92 | 1.07 |
| Small cities | 1.17 | 1.13 | 1.20 |  | 1.11 | 1.06 | 1.15 |  | 1.07 | 1.00 | 1.13 |  | 0.99 | 0.92 | 1.07 |
|  |  |  |  |  |  |  |  |  |  |  |  |  |  |  |  |
| Cardiovascular disease |  |  |  |  |  |  |  |  |  |  |  |  |  |  |  |
| No | 1.00 | Reference | |  | 1.00 | Reference | |  | 1.00 | Reference | |  | 1.00 | Reference | |
| Yes | 0.78 | 0.75 | 0.80 |  | 0.60 | 0.59 | 0.62 |  | 1.28 | 1.22 | 1.35 |  | 0.99 | 0.94 | 1.05 |
|  |  |  |  |  |  |  |  |  |  |  |  |  |  |  |  |
| Diabetes |  |  |  |  |  |  |  |  |  |  |  |  |  |  |  |
| No | 1.00 | Reference | |  | 1.00 | Reference | |  | 1.00 | Reference | |  | 1.00 | Reference | |
| Yes | 0.78 | 0.71 | 0.86 |  | 0.78 | 0.71 | 0.85 |  | 0.92 | 0.78 | 1.08 |  | 0.99 | 0.85 | 1.15 |
|  |  |  |  |  |  |  |  |  |  |  |  |  |  |  |  |
| Rheumatoid arthritis/systemic lupus erythematosus | |  |  |  |  |  |  |  |  |  |  |  |  |  |  |
| No | 1.00 | Reference | |  | 1.00 | Reference | |  | 1.00 | Reference | |  | 1.00 | Reference | |
| Yes | 1.44 | 1.29 | 1.60 |  | 1.62 | 1.45 | 1.80 |  | 3.48 | 3.03 | 4.01 |  | 3.65 | 3.16 | 4.21 |
|  |  |  |  |  |  |  |  |  |  |  |  |  |  |  |  |
| Chronic obstructive pulmonary disease/asthma |  |  |  |  |  |  |  |  |  |  |  |  |  |  |  |
| No | 1.00 | Reference | |  | 1.00 | Reference | |  | 1.00 | Reference | |  | 1.00 | Reference | |
| Yes | 1.28 | 1.18 | 1.38 |  | 1.04 | 0.97 | 1.11 |  | 2.16 | 1.92 | 2.43 |  | 1.48 | 1.32 | 1.65 |
